# A region-wide hub and spoke approach to standardize and decentralize type 1 diabetes management

**DOI:** 10.3389/fpubh.2026.1763175

**Published:** 2026-02-11

**Authors:** Nicola Minuto, Giordano Spacco, Giulia Siri, Massimiliano Leoni, Alberto Gaiero, Maria Franca Corona, Diego Minghetti, Luca Antonio Ramenghi, Andrea Pintabona, Maria Grazia Calevo, Marisa Alberti, Raffaele Spiazzi, Giuseppe Spiga, Marta Bassi, Maghnie Mohamad

**Affiliations:** 1 Pediatric Clinic, Department of Pediatric Clinical Sciences IRCCS Istituto Giannina Gaslini, Genoa, Italy; 2 Department of Neuroscience, Rehabilitation, Ophthalmology, Genetics, Maternal and Child Health (DINOGMI), University of Genoa, Genoa, Italy; 3 Pediatrics and Neonatology Unit of Savona, IRCCS Istituto Giannina Gaslini, Genoa, Italy; 4 Pediatrics and Neonatology Unit of La Spezia, IRCCS Istituto Giannina Gaslini, Genoa, Italy; 5 Pediatrics and Neonatology Unit of Imperia, IRCCS Istituto Giannina Gaslini, Imperia, Italy; 6 Neonatal Intensive Care Unit, Department of Maternal and Neonatal Health, IRCCS Istituto Giannina Gaslini, Genoa, Italy; 7 Psychology Unit, IRCCS Istituto Giannina Gaslini, Genoa, Italy; 8 Biostatistics Unit, Scientific Directorate, IRCCS Istituto Giannina Gaslini, Genoa, Italy; 9 Healthcare Directorate, IRCCS Istituto Giannina Gaslini, Genoa, Italy

**Keywords:** environmental sustainability, healthcare accessibility, hub, spoke, type 1 diabetes

## Abstract

**Background and aims:**

Type 1 diabetes (T1D) requires specialized, multidisciplinary, and technology-driven care, which can be difficult to guarantee across regions with fragmented services. In July 2023, the Liguria region (Italy) reorganized pediatric diabetes care into a Hub and Spoke model, coordinated by the Gaslini Institute (hub) and three peripheral pediatric departments (spokes). This study aimed to evaluate the first year of application of this model in terms of clinical, technological, and environmental outcomes.

**Methods:**

This retrospective observational study included T1D patients aged less than 35 years in follow-up at the new regional Hub and Spoke system. Patients were categorized as transferred (previously followed at the hub), acquired (previously followed at a local center), and new onset (followed from the beginning at the Hub and Spoke system). The primary outcome was the change in time in range 70–180 mg/dL after 12 months. Secondary outcomes were additional indicators of glycemic control (CGM metrics), adoption of technologies (rtCGM and AID), and economic and environmental impact (travel distance, costs, and CO₂ emissions).

**Results:**

129 patients were referred to the new Hub and Spoke system during the first 2 years of its implementation. Among them, 88 (50 transferred, 38 acquired) had available data at baseline and completed the 1-year follow up period and were included in the study. In this cohort, TIR increased from 57.2 to 65.1% and TITR from 35.1 to 41.7% (*p* < 0.001), with improvement in other CGM metrics. Improvements were more pronounced in acquired patients, whose TIR rose from 53.9 to 65.7% (+11.2%, *p* < 0.001). AID use increased significantly with a larger rise among acquired (from 26.3 to 60.5%, +34.2%) compared with transferred patients (+26%). The program generated environmental and economic benefits, with estimated 70,192 km of travel, 11,932 kg CO₂, and € 25,760 saved yearly.

**Conclusion:**

The Hub and Spoke model proved feasible and effective, leading to more standardized care delivery, broader adoption of diabetes technologies, improved glycemic control, and significant reductions in travel-related costs and environmental impact. This approach may offer a scalable and sustainable solution for managing T1D, particularly in regions with complex geography and variable healthcare infrastructures.

## 1 Introduction

Type 1 diabetes (T1D) is one of the most common chronic diseases in childhood, and its incidence has been steadily increasing worldwide (1). In Italy, the estimated prevalence of T1D is approximately 0.5% in the general population and 0.22% in the pediatric population, with about 12.3 new cases per 100,000 children per year (2). The Liguria region, in northwestern Italy, has reported an incidence of pediatric T1D of 12.56 per 100,000 children per year, in line with the national average (3). Based on data of our center from the past 5 years and current demographic estimates, the annual pediatric incidence appears substantially unchanged, at approximately 14 per 100,000 children per year. Given the clinical complexity of this condition, which requires lifelong follow-up and frequent therapeutic adjustments, ensuring equitable access to expert pediatric diabetes care across the region is a major public health priority (4). According to the 2022 ISPAD guidelines, pediatric diabetes care should be person-centered, placing families at the core of the care team, while also ensuring continuous integration of the most advanced technologies into treatment plans and guaranteeing access to multidisciplinary expertise, either locally or, when not available, through telemedicine support from regional specialized centers (5). Liguria is divided into four provinces: Genoa, La Spezia, Imperia, and Savona. In Genoa, the regional tertiary care hospital, “IRCCS Istituto Giannina Gaslini” (IGG), hosts the reference center specialized in the management of pediatric T1D. In the other provinces, pediatric diabetes care was historically provided within general pediatric departments, each under the administration of its respective local health authority. This organizational structure resulted in hospitals operating independently from one another, leading to heterogeneous clinical practices. For instance, some hospitals referred all newly diagnosed patients to the regional center in Genoa, while others transferred only selected cases, such as those presenting with severe diabetic ketoacidosis (DKA). Protocols for the management of DKA also varied between institutions. After the initial diagnosis, some families living outside Genoa opted to continue follow-up at the tertiary center, accepting the time and travel burden involved, whereas others chose local care within their home hospitals. This scenario changed significantly in July 2023, when all pediatric departments in Liguria were officially integrated under the management of IGG, establishing a unified regional governance model. As part of this reorganization, a standardized care protocol for pediatric T1D was developed through a collaborative process involving pediatric diabetologists and the medical directorate of the regional reference center, together with the heads of the peripheral pediatric departments. The protocol clearly defines the roles and responsibilities of all professionals involved in the care of children with T1D and is available on the institutional intranet and provided as Supplementary material. The new model was based on a regional Hub and Spoke organization, in which all children and adolescents with T1D in Liguria are taken in charge within the IGG network, regardless of their place of diagnosis. The Hub and Spoke organization design has been traditionally described as a network in which a central hub concentrates specialized expertise and resources, while peripheral spokes provide care locally and connect patients to the hub when more advanced services are required (6). Within the context of the Gaslini experience in pediatric T1D care the hub is represented by the IGG Regional Reference Center for Pediatric Diabetes (Genoa), while the spokes are the pediatric departments of the three peripheral hospitals (Imperia, Savona, and La Spezia). This reorganization aimed to guarantee high-quality follow-up for all children and adolescents with T1D as close as possible to their homes, in line with international guidelines (4, 5). Shared standards of care have therefore been implemented across the network to ensure consistency in chronic management, therapeutic education, and the use of advanced technologies such as real-time Continuous Glucose Monitoring (rtCGM) and Automated Insulin Delivery (AID) systems. This model also facilitated equitable access to multidisciplinary expertise considered essential for pediatric diabetes care (7), particularly for patients previously followed exclusively locally, through structured referral pathways to the Hub. In addition, the project has also aimed to harmonize the approach to new-onset T1D: shared protocols for the management of cases with and without diabetic ketoacidosis have been developed, together with shared criteria defining which patients can be safely managed at the spoke level and which require transfer to the hub. The aim of this study was to evaluate the clinical and organizational impact of the first year of implementation of this Hub and Spoke model in Liguria.

## 2 Methods

### 2.1 Description of the regional hub-and-spoke model

**Figure 1:**
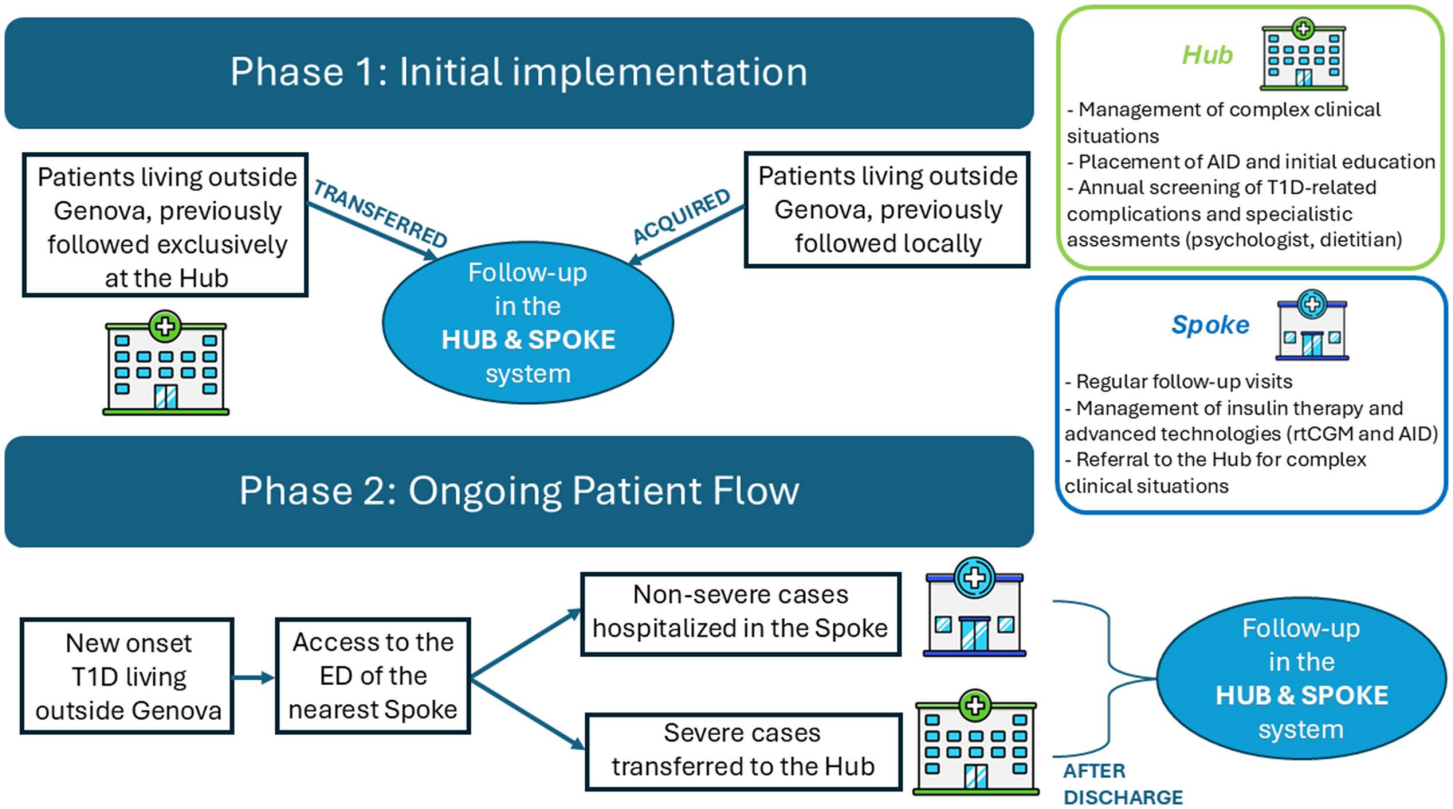
Patient entry pathways and care organization within the regional Hub and Spoke model. At the time of model implementation (Phase 1), patients living outside Genoa, previously followed exclusively at the Hub were offered the possibility to continue follow-up at the spoke center closest to their residence (transferred patients), while patients previously followed locally were newly taken in charge within the Gaslini network while maintaining follow-up at their local spoke (acquired patients). During the ongoing operational phase, patients with new-onset T1D present to the ED of the nearest spoke hospital. According to predefined and shared clinical and laboratory criteria, severe cases are transferred to the Hub, whereas non-severe cases are managed locally at the spoke. After discharge, routine follow-up is provided at the spoke center closest to the patient’s home. Green and blue boxes summarize the main functions of the Hub and Spokes, respectively, within the regional care network. ED, emergency department; T1D, type 1 diabetes; AID, Automated Insulin Delivery systems; rtCGM, real-time Continuous Glucose Monitoring.

Patient entry pathways within the Hub and Spoke system are illustrated in Figure 1 and differ between the initial implementation phase and the ongoing operational phase. Routine follow-up care is delivered at spoke centers through joint outpatient clinics conducted by a pediatric diabetologist from the hub together with a local hospital pediatrician designated as diabetes referent. Follow-up visits are scheduled on average every 3 months and focus on the assessment of glycemic control, therapeutic adjustments, and reinforcement of diabetes self-management education. Outside scheduled visits, local diabetes referents remain available to provide additional support and unscheduled visits when required. Spoke centers are equipped to manage modern diabetes technologies, including rtCGM and AID systems. This capability is ensured through the presence of a pediatric diabetologist from the Hub during joint clinics and through regular training sessions organized by hub specialists, which are addressed not only to designated local diabetes referents but also to the broader medical and nursing staff of spoke centers, thereby ensuring continuity of care in settings characterized by shift-based organization. Routine device-related issues, such as consumable replacement, pump or sensor troubleshooting, and therapy reprogramming, are managed at the spoke level, with support from hub specialists when needed. Communication between hub and spoke professionals is ensured through direct contacts and telemedicine tools when appropriate. In the absence of complications or specific clinical needs, patients attend the hub once per year for a comprehensive day-hospital evaluation, including screening for autoimmune comorbidities, chronic complications, and psychological and nutritional assessments. Additional hub-based evaluations are scheduled when clinically indicated and for the management of the most complex clinical cases.

### 2.2 Study design and population

This retrospective observational study was conducted within a Hub and Spoke model coordinated by the IGG, which manages both the regional reference center for pediatric diabetes in Genoa (hub) and three peripheral pediatric departments in Imperia, Savona, and La Spezia (spokes). The study inclusion period extended from July 1, 2023, to June 30, 2025, corresponding to the first 2 years of implementation of the model. Eligible participants were all individuals younger than 35 years with T1D, defined according to ADA criteria (8), and who were taken in charge within the new Hub and Spoke model during the inclusion period. Although the Hub-and-Spoke model is pediatric-led, young adult patients up to 35 years of age were also included to reflect local clinical practice, in which continuity of diabetes care is ensured beyond adolescence, and to capture the overall organizational workload of the system. For descriptive purposes, patients were further classified into three categories reflecting their mode of entry into the Hub and Spoke system:

- Transferred patients: previously followed exclusively at the hub and now taken in charge for follow-up at a spoke center of the Hub and Spoke system.
- Acquired patients: patients previously followed locally but taken in charge at a spoke center after the implementation of the Hub and Spoke system.
- New-onset patients: newly diagnosed patients with T1D taken in charge and followed from the beginning at a spoke center within the Hub and Spoke system. This group was considered exclusively for the assessment of the overall organizational workload of the Hub and Spoke system during its first 2 years of implementation.

The study was conducted in compliance with the Declaration of Helsinki and the International Conference on Harmonization Good Clinical Practice guidelines. All data were anonymized prior to analysis, and written informed consent had been obtained from patients or their guardians at the time of care. Ethical review and approval were not required for this study in accordance with Italian regulations and institutional requirements and this exemption from formal ethical review was confirmed by the institutional authorities of IRCCS Istituto Giannina Gaslini. Under Italian law (Italian Data Protection Authority Authorization No. 9/2014), retrospective studies using fully anonymized clinical data do not require additional ethical approval.

### 2.3 Outcomes and data collection

The primary outcome of the study was the change in glycemic control, assessed as time in range 70–180 mg/dL (TIR) after 1 year of follow-up at a spoke center within the Hub and Spoke system. The following patients were excluded from this analysis: patients in the new-onset group, since no baseline comparison was possible, patients with less than 12 months of follow-up and those with absent or insufficient CGM data, defined as fewer than 12 days of valid recordings or <80% sensor usage in the predefined assessment window.

Secondary outcomes included:

- Additional indicators of glycemic control, measured as the change between T0 and T1 of CGM-derived metrics: time in tight range 70–140 mg/dL (TITR), time above range 181–250 mg/dL (TAR), time above 250 mg/dL (TAR250), time below range 54–69 mg/dL (TBR), time below 54 mg/dL (TBR54), average glucose (AG), standard deviation (SD), coefficient of variation (CV), glucose management indicator (GMI), glycemia risk index (GRI) and percentage of CGM use (%CGM).
- Adoption of advanced technologies, measured as the change between T0 and T1 in the proportion of patients using real-time continuous glucose monitoring (rtCGM) and automated insulin delivery (AID) systems. AID was defined as any pump able to adjust insulin delivery in response to sensor glucose data (9). Glucose monitoring was categorized as self-monitoring of blood glucose (SMBG), flash glucose monitoring (FGM), or real-time CGM (rtCGM). Insulin therapy was classified as multiple daily injections (MDI), sensor-augmented pump (SAP), pumps with pre-low glucose suspend (PLGS), or AID systems.
- Clinical parameters, including changes in BMI and BMI z-score.
- Service utilization outcomes, assessed as the number of outpatient visits performed in the year preceding each timepoint.
- Environmental outcomes, expressed in terms of kilometers saved, CO₂ emissions spared, and travel-related costs avoided. Environmental outcomes were estimated through travel analyses. Using ViaMichelin® route planning, we calculated the round-trip distance between each spoke center and the regional hub in Genoa (10). For each patient, the number of visits performed at the spoke during the 1-year follow-up period was multiplied by the corresponding round-trip distance, under the assumption that patients lived near their local center. CO₂ emissions spared were obtained by multiplying kilometers saved by the national average emission factor for private vehicles in Italy, as reported by ISPRA (0.17 kgCO₂/km) (11). Travel-related cost savings were estimated using the official per-kilometer operating cost published in the 2025 ACI (Automobile Club d’Italia) tables (12). A Fiat Panda 1.2 petrol (69 CV), the most widely owned city car in Italy, was selected as the reference vehicle, with a reported cost of €0.367/km.

Clinical, glycemic, environmental and service utilization data were collected at baseline (T0), corresponding to the first outpatient visit performed at a spoke center, and at the 12-month (±3 months) follow-up visit (T1). Information on number of outpatient visits in the previous 12 months were available at baseline only for transferred patients due to lack of pre-implementation data for acquired patients. All data were recorded in the shared electronic platform REDCap® (Vanderbilt University, Nashville, TN, United States).

### 2.4 Statistical analysis

Descriptive statistics were generated for the whole cohort and data were expressed as mean and standard deviation (SD), median and range for continuous variables and as absolute or relative frequencies for categorical variables. Statistical comparisons were performed using the paired Wilcoxon signed-rank test for continuous variables and χ2 or Fisher’s exact test for categorical variables. Effect sizes were quantified using Cohen’s d (small effect: 0.20–0.49; medium effect: 0.50–0.79; large effect: ≥0.80) (13). A *p* value less than 0.05 was considered statistically significant, and all *p* values were based upon two tailed tests. Statistical analysis was performed using SPSS for Windows (IBM SPSS Inc., New York, NY, United States).

## 3 Results

### 3.1 Study population and baseline characteristics

**Figure 2:**
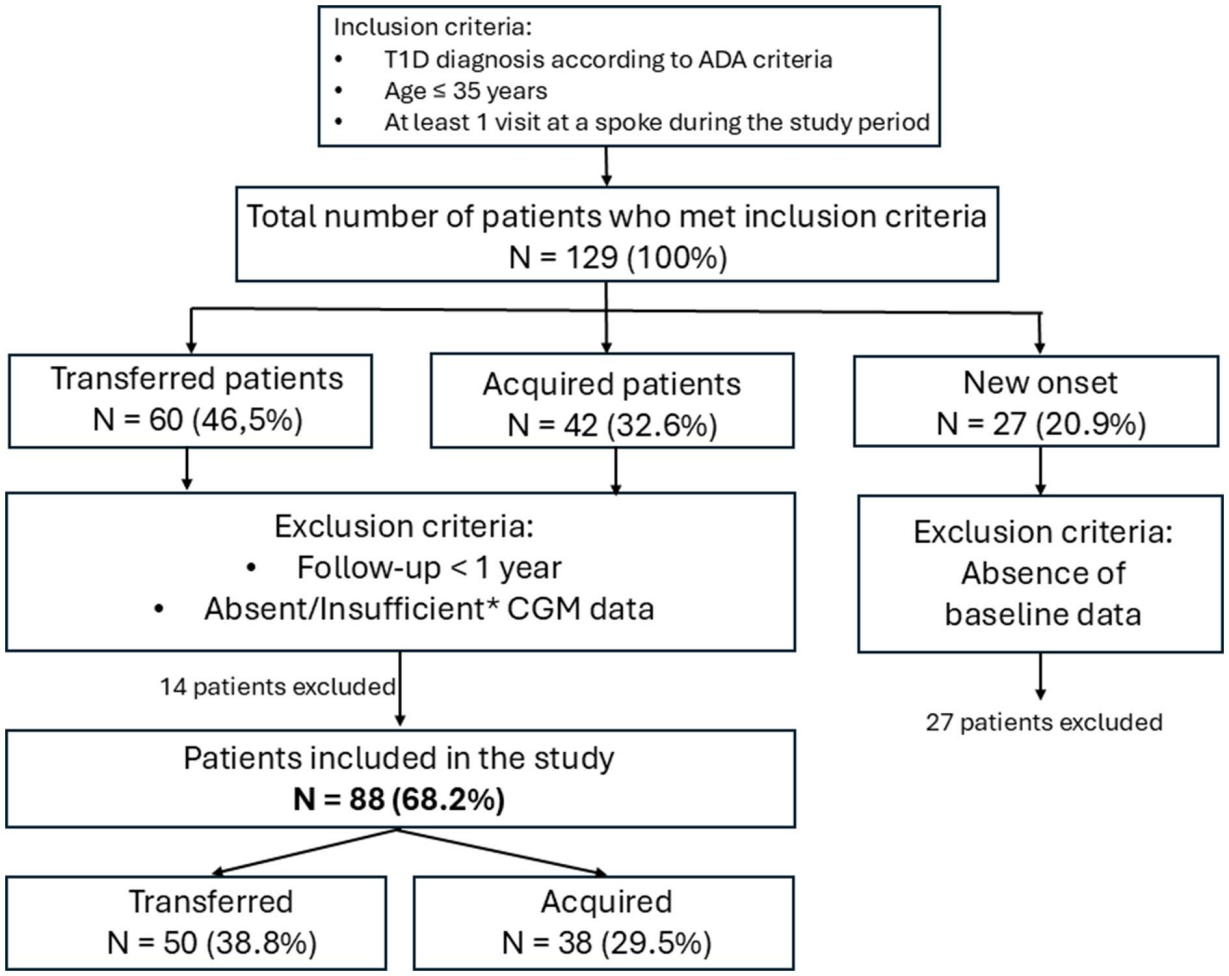
Flow of participants through the study. All percentages shown in the figure refer to the total number of patients managed within the Hub and Spoke model during its first 2 years of implementation (*N* = 129). *CGM data are considered insufficient when <12 days of valid recordings or <80% sensor usage were available in the 14 days before each time point. T1D, Type 1 Diabetes; ADA, American Diabetes Association.

During the first 2 years of implementation, 129 patients were referred to the new Hub and Spoke system, of whom 27 were new-onset patients and 102 were previously followed patients. Clinical and demographic characteristics of these patients are presented in Supplementary Tables 1, 2, respectively. Of all patients enrolled in the Hub and Spoke system, 88 had complete data available at baseline and at 1 year follow-up and were included in the study. The flow of the selection of study population is shown in Figure 2. Baseline characteristics of the patients included in the study are presented in Table 1. The mean age at T0 was 13 years ± 5.9 (range 3–35 years), with an average disease duration of 10.2 years ± 7.1. Males represented 53.4% of the cohort, and most patients (80.7%) were Caucasian, followed by individuals of African ethnicity (17.1%). 50 patients (56.8%) were transferred (previously followed exclusively at the hub and then taken in charge at a spoke center of the Hub and Spoke system) and 38 patients (43.2%) were acquired (previously followed locally and then taken in charge at a spoke center of the Hub and Spoke system). Patients were evenly distributed across the three spokes (38.6, 37.5 and 23.9%, respectively).

**Table 1:** Baseline characteristics of patients included in the study (*N* = 88).

Variable	T0-Baseline (*N* = 88)
Sex assigned at birth	
Female	41 (46.6%)
Male	47 (53.4%)
Ethnicity	
Asiatic	1 (1.1%)
African	15 (17.1%)
Hispanic	1 (1.1%)
Caucasic	71 (80.7%)
Previous care setting	
Transferred	50 (56.8%)
Acquired	38 (43.2%)
Current Spoke Center	
Imperia	34 (38.6%)
La Spezia	21 (23.9%)
Savona	33 (37.5%)
Age (years)	13 ± 5.9
Disease duration (years)	10.2 ± 7.1

Data are presented as mean ± SD or as n (%), unless otherwise specified.

### 3.2 Follow-up data (12-month)

**Figure 3:**
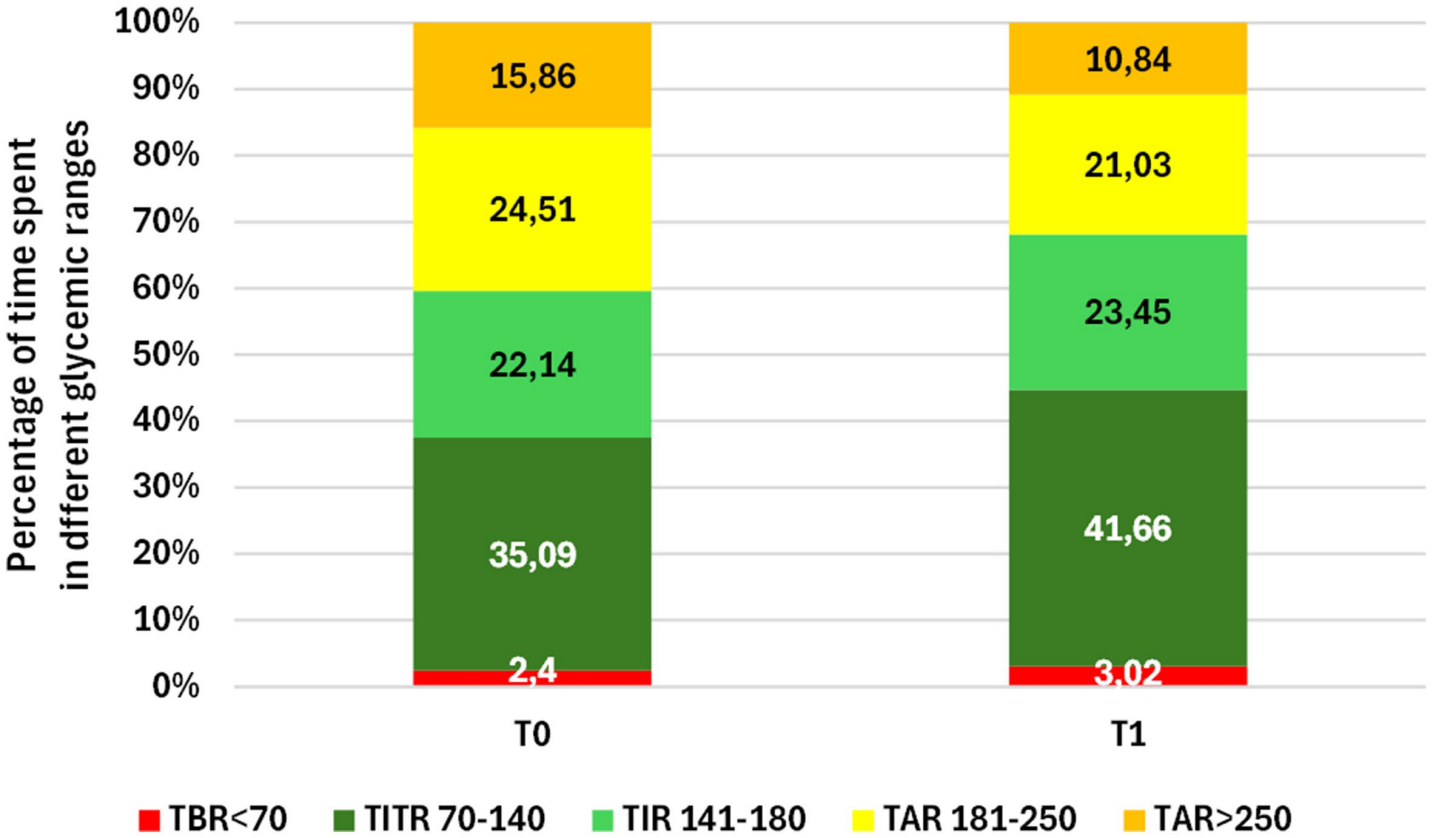
Changes in glycemic control, assessed through percentage of time spent in different CGM ranges, at baseline (T0) and after 1 year of follow-up at a spoke center of the Hub and Spoke system (T1) in the 88 patients included in the study.

**Figure 4:**
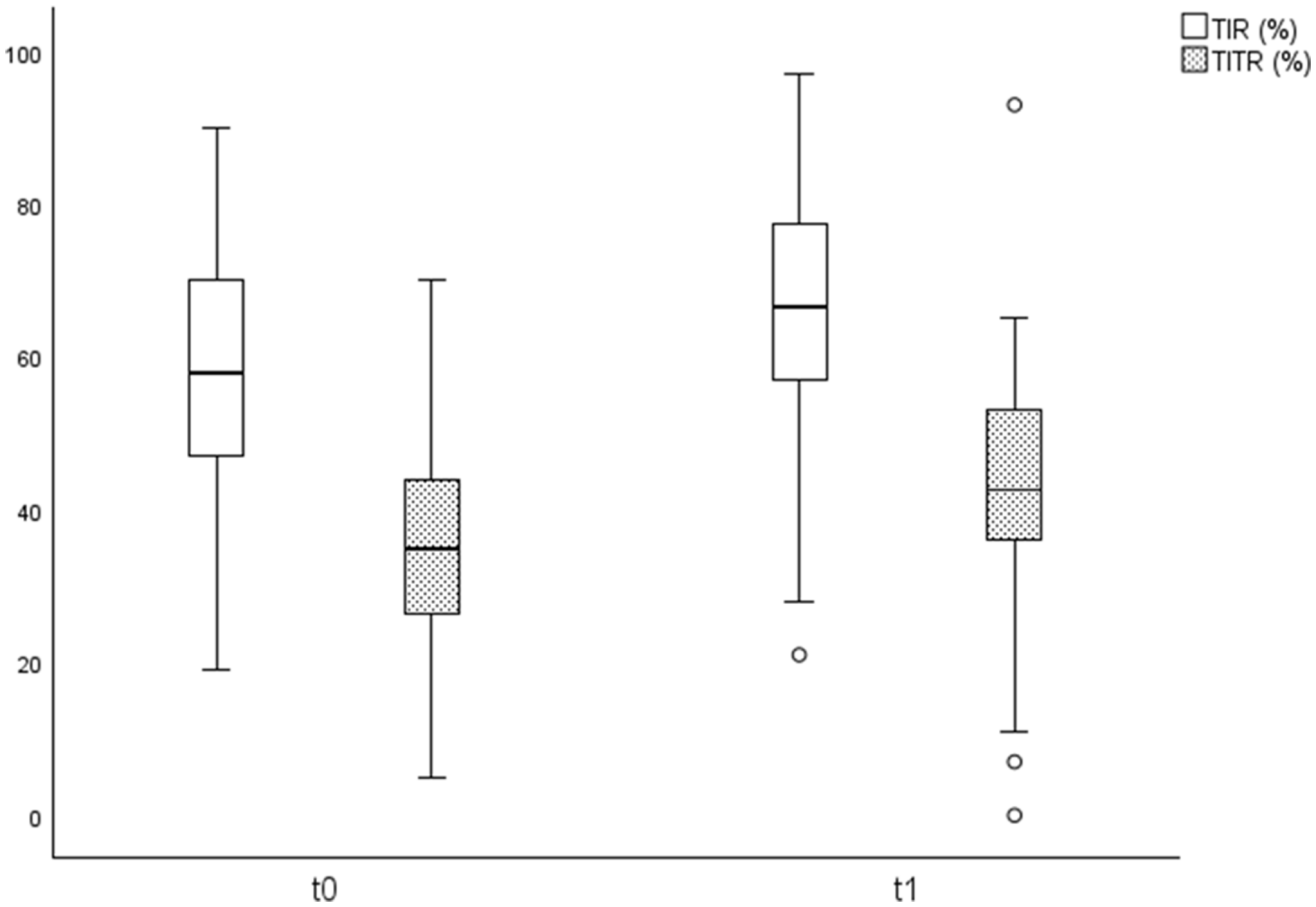
Improvements in time in range 70–180 mg/dL (TIR) and time in tight range 70–140 mg/dL (TITR) at baseline (T0) and after 1 year of follow-up at a spoke center of the Hub and Spoke system (T1) in the 88 patients included in the study.

**Figure 5:**
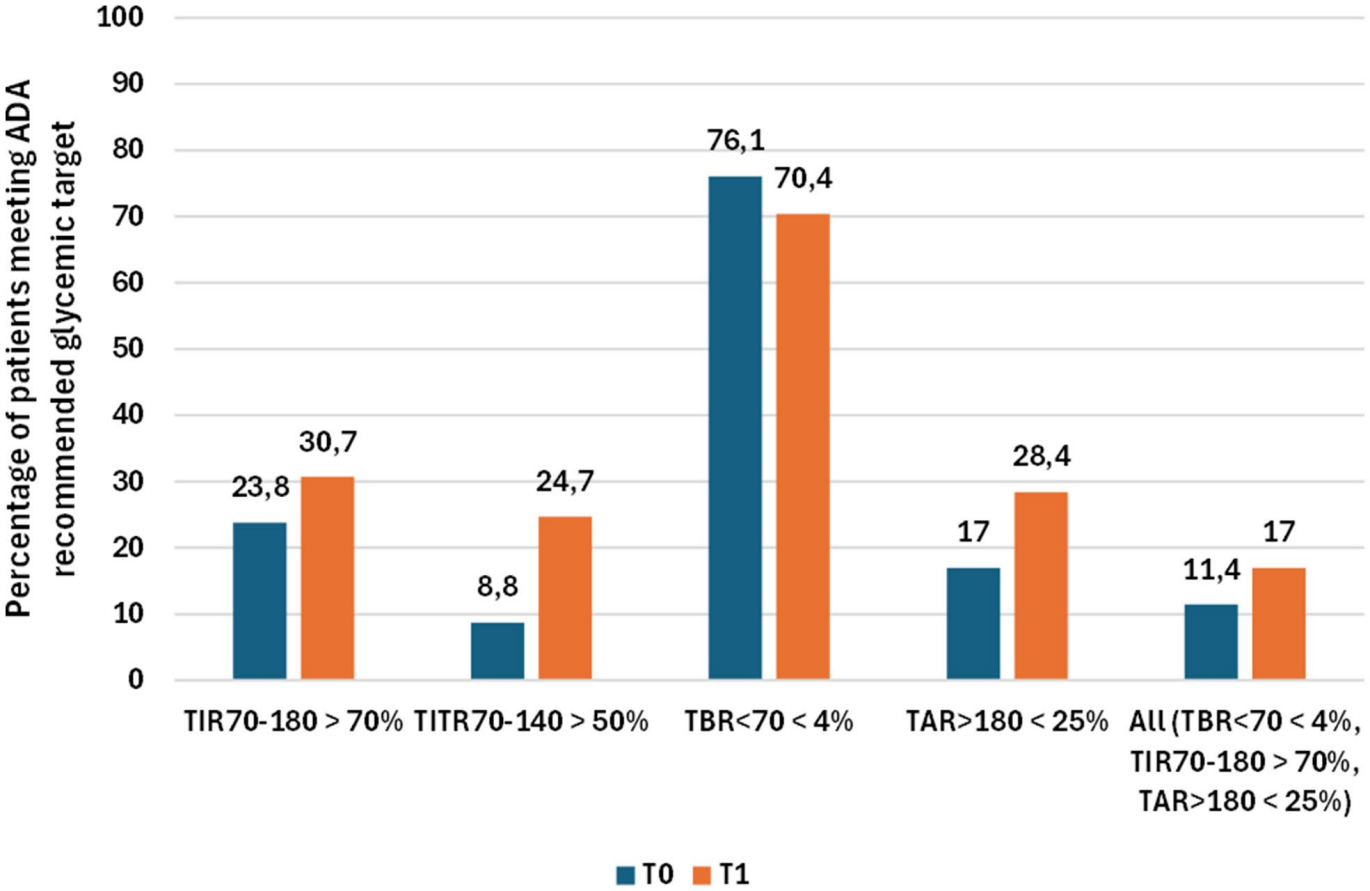
Percentage of patients meeting ADA-recommended glycemic targets at baseline (T0) and after 12 months of follow-up (T1) in the 88 patients included in the study. For each glycemic target, paired columns represent the proportion of patients achieving the recommended threshold at T0 and T1.

Among transferred and acquired patients, 88 had available data at T0 and T1. A statistically significant improvement in glycemic control was observed in this cohort (Table 2; Figure 3). Time in Range 70–180 mg/dL (TIR) increased from 57.23 to 65.13%, and Time in Tight Range 70–140 mg/dL (TITR) from 35.09 to 41.66% (both *p* < 0.001), with parallel reductions in Time above Range 181–250 mg/dL (TAR) and Time above 250 mg/dL (TAR250; *p* < 0.001). Average Glucose (AG) declined from 176.13 to 163.15 mg/dL, with corresponding reductions in Glucose management Indicator (GMI; from 7.51 to 7.20%) and Glycemia Risk Index (GRI), that decreased from 51.44 to 42.13 (p < 0.001 for all the variables). Time below 54 mg/dL (TBR54) increased slightly, from 0.52 to 0.77% (*p* = 0.03), but remained within recommended thresholds, while Time below Range 54–69 mg/dL (TBR) showed no significant changes. Effect size analysis showed medium effects for TIR (Cohen’s d = 0.54) and medium effects for TITR (d = 0.51; Figure 4). Accordingly, the percentage of patients meeting ADA-recommended targets for Time in Range and Time in Tight Range increased at 12 months compared with baseline, from 23.8 to 30.7% and from 8.8 to 24.7%, respectively (Figure 5) (14). No significant changes were observed in BMI, BMI z-score, Coefficient of Variation (CV) or sensor usage over 12 months in the overall cohort.

**Table 2:** Clinical and glycemic outcomes at baseline (T0) and after 12 months of follow-up (T1) in the 88 patients included in the study.

Variable	T0	T1	*p* value
TBR54 (%)	0.52 ± 1.19	0.77 ± 1.61	**0.03**
TBR (%)	1.88 ± 1.86	2.25 ± 2.30	0.25
TIR (%)	57.23 ± 16.12	65.13 ± 13.17	**<0.001**
TITR (%)	35.09 ± 13.71	41.66 ± 11.77	**<0.001**
TAR (%)	24.51 ± 7.07	21.03 ± 6.36	**<0.001**
TAR250 (%)	15.86 ± 11.90	10.84 ± 9.04	**<0.001**
AG (mg/dL)	176.13 ± 29.33	163.15 ± 23.09	**<0.001**
SD (mg/dL)	66.65 ± 16.17	62.13 ± 15.38	**0.01**
CV (%)	37.72 ± 6.16	37.97 ± 7.26	0.98
GMI (%)	7.51 ± 0.70	7.2 ± 0.55	**<0.001**
%CGM (%)	95.51 ± 4.51	94.94 ± 6.59	0.40
GRI (%)	51.44 ± 21.83	42.13 ± 18.64	**<0.001**
BMI	19.77 ± 3.66	20.09 ± 0.74	0.33
BMI z-score	0.27 ± 1.22	0.30 ± 1.04	0.54

Values are presented as mean ± SD. TBR54, time below range < 54 mg/dL; TBR, time below range 54–69 mg/dL; TIR, time in range 70–180 mg/dL; TITR, time in tight range 70–140 mg/dL; TAR, time above range 181–250 mg/dL; TAR250, time above range > 250 mg/dL; AG, average glucose; SD, standard deviation; CV, coefficient of variation; GMI, glucose management indicator; %CGM, percentage use of CGM, GRI, glycemia risk index; HbA1c, glycated hemoglobin; BMI, body mass index. Bold means statistically significant.

### 3.3 Comparative analysis

Stratified analysis revealed greater improvement in acquired than in transferred patients. As shown in Table 3, among acquired patients TIR increased from 53.97 to 65.74% and TITR from 32.72 to 43.34% (both *p* < 0.001) in association with marked reductions in AG (from 182.36 to 158.67 mg/dL, *p* < 0.001), GMI (from 7.63 to 7.10%, *p* = 0.01) and GRI (from 54.87 to 42.68, *p* = 0.002). However, in this group, TBR increased significantly from 1.74 to 3.21% (*p* = 0.004). Effect size analysis showed large effects for TIR (Cohen’s d = 1.23) and large effects for TITR (d = 1.25). Transferred patients, who had better baseline glycemic control, showed smaller improvements in glycemic metrics over time (Table 4). Effect size analysis showed small effects for TIR (Cohen’s d = 0.45) and small effects for TITR (d = 0.40).

**Table 3:** Glycemic and organizational outcomes at baseline (T0) and after 12 months of follow-up (T1) in the subgroup of acquired patients (*n* = 38).

Variable	T0	T1	*p* value
TBR54 (%)	0.34 ± 0.53	1.32 ± 2.26	**0.002**
TBR (%)	1.74 ± 1.43	3.21 ± 2.79	**0.004**
TIR (%)	53.97 ± 16.42	65.74 ± 12.60	**<0.001**
TITR (%)	32.72 ± 14.59	43.34 ± 11.31	**<0.001**
TAR (%)	25.71 ± 7.06	19.95 ± 6.62	**<0.001**
TAR250 (%)	18.24 ± 12.23	9.84 ± 7.38	**<0.001**
AG (mg/dL)	182.36 ± 30.10	158.67 ± 2.66	**<0.001**
SD (mg/dL)	68.94 ± 17.49	63.85 ± 15.16	0.11
CV (%)	37.46 ± 5.77	40.18 ± 8.02	**0.04**
GMI (%)	7.63 ± 0.72	7.10 ± 0.50	**0.01**
%CGM (%)	95.30 ± 4.49	95.08 ± 8.89	0.33
GRI (%)	54.87 ± 21.51	42.68 ± 18.39	**0.002**
N. of visits*	n.a	6.38 ± 1.77	

Values are presented as mean ± SD. TBR54, time below range < 54 mg/dL; TBR, time below range 54–69 mg/dL; TIR, time in range 70–180 mg/dL; TITR, time in tight range 70–140 mg/dL; TAR, time above range 181–250 mg/dL; TAR250, time above range > 250 mg/dL; AG, average glucose; SD, standard deviation; CV, coefficient of variation; GMI, glucose management indicator; %CGM, percentage use of CGM, GRI, glycemia risk index; HbA1c, glycated hemoglobin; n.a., not available. Bold means statistically significant. *Number of outpatient visits is evaluated over the 12-month period preceding each timepoint. Data at baseline were not available for acquired patients previously followed outside the Gaslini network.

**Table 4:** Glycemic and organizational outcomes at baseline (T0) and after 12 months of follow-up (T1) in the subgroup of transferred patients (*n* = 50).

Variable	T0	T1	*p* value
TBR54 (%)	0.66 ± 1.51	0.36 ± 0.60	0.13
TBR (%)	1.98 ± 2.13	1.52 ± 1.50	0.14
TIR (%)	59.70 ± 15.59	64.66 ± 13.69	**0.007**
TITR (%)	36.70 ± 12.99	40.51 ± 12.05	**0.04**
TAR (%)	23.60 ± 7.02	21.86 ± 6.10	**0.05**
TAR250 (%)	14.06 ± 11.43	11.60 ± 10.13	0.09
AG (mg/dL)	171.74 ± 28.28	166.30 ± 24.38	0.13
SD (mg/dL)	65.04 ± 15.17	60.91 ± 15.58	**0.03**
CV (%)	37.90 ± 6.47	36.42 ± 6.31	0.06
GMI (%)	7.42 ± 0.68	7.26 ± 0.58	0.10
%CGM (%)	95.65 ± 4.56	94.85 ± 4.43	**0.04**
GRI (%)	48.78 ± 21.92	41.69 ± 19.01	**0.02**
N. of visits*	5.33 ± 1.99	5.60 ± 1.95	0.32

Values are presented as mean ± SD. TBR54, time below range < 54 mg/dL; TBR, time below range 54–69 mg/dL; TIR, time in range 70–180 mg/dL; TITR, time in tight range 70–140 mg/dL; TAR, time above range 181–250 mg/dL; TAR250, time above range > 250 mg/dL; AG, average glucose; SD, standard deviation; CV, coefficient of variation; GMI, glucose management indicator; %CGM, percentage use of CGM, GRI, glycemia risk index; HbA1c, glycated hemoglobin. Bold means statistically significant. *Number of outpatient visits is evaluated over the 12-month period preceding each timepoint.

**Figure 6:**
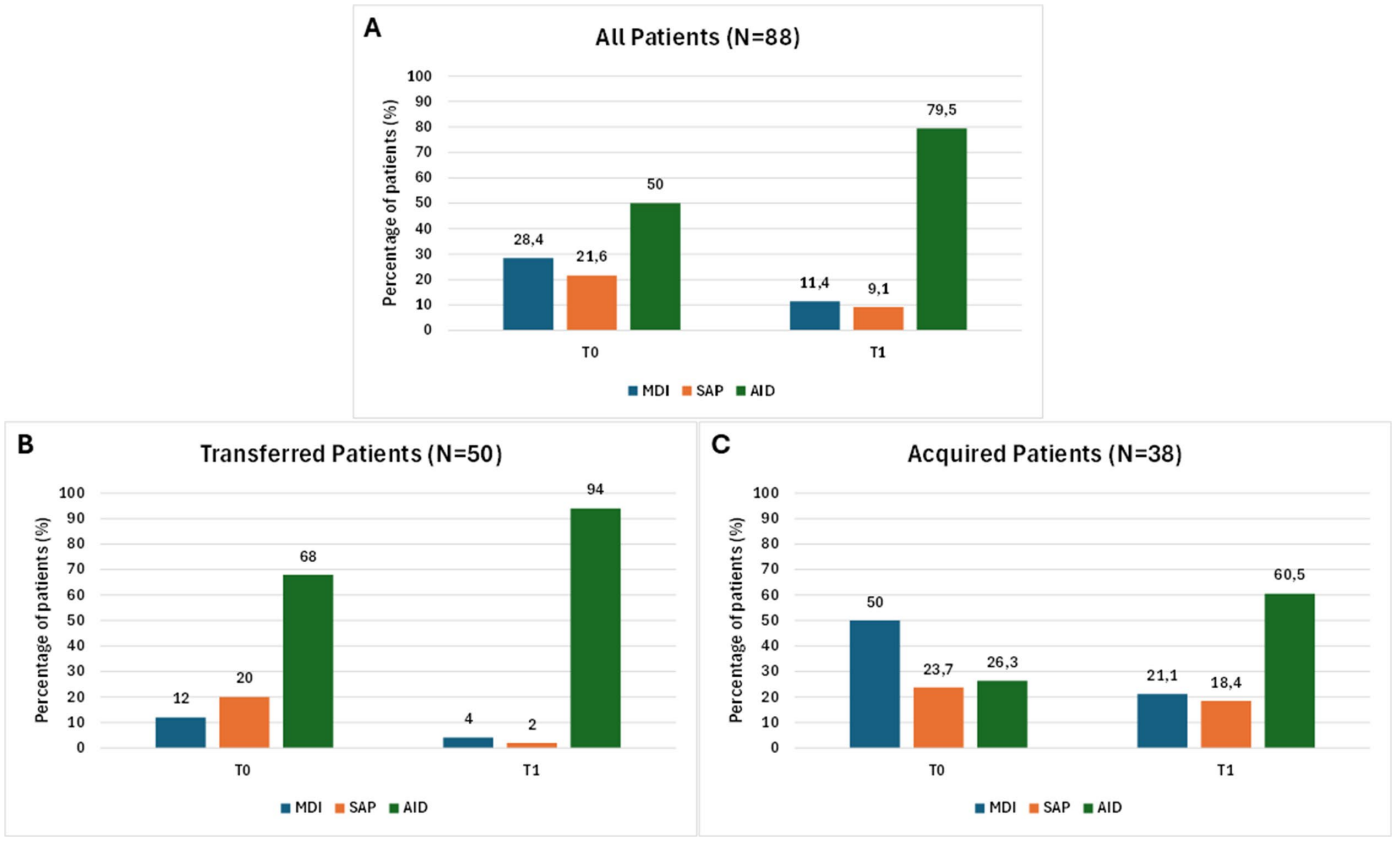
Changes in insulin therapy over the study period. Bars represent the percentage of patients in each treatment category at baseline (T0) and after 12 months of follow-up (T1). Panel (**A**): overall cohort (*n* = 88); Panel (**B**): transferred patients (*n* = 50); Panel (**C**): acquired patients (*n* = 38). MDI, Multiple Daily Injection; SAP, Sensor Augmented Pump; AID, Automated Insulin Delivery.

The overall improvement in glycemic control paralleled the increased use of advanced technologies during follow-up (Table 5). At baseline, 88.6% of patients were using rtCGM, while the remaining 11.4% used an FGM. Regarding insulin therapy, 50.0% of patients were treated with an AID system, 28.4% were on MDI, 21.6% on SAP therapy, while none of the participants were under therapy with a PLGS system. At baseline, the use of advanced technologies was substantially higher among transferred patients, with 68.0% using AID and 100% using rtCGM, compared with 26.3 and 76.7%, respectively, in acquired patients. As shown in Figure 6, in the 88 patients included in the comparative analysis, AID use rose from 50 to 79.5%, with a larger increase among acquired patients (+34.2%, *p* = 0.005) compared with transferred patients (+26.0%, *p* = 0.002), with statistically significant increases observed in both subgroups. rtCGM systems use also significantly increased from 73.7 to 97.4% in acquired patients (*p* = 0.007), while transferred patients maintained a rtCGM use of 100% throughout the study. In transferred patients, for whom longitudinal data were available, the number of outpatient visits per year did not change significantly over 12 months.

**Table 5:** Use of technologies at baseline and 1 year after the implementation of the regional Hub and Spoke model.

Variable	T0	T1	% variation	*p* value
All patients (*N* = 88)				
MDI	25 (28.4%)	10 (11.4%)	−17%	
SAP	19 (21.6%)	8 (9.1%)	−12.5%	
AID	44 (50%)	70 (79.5%)	+29.5%	
FGM	10 (11.4%)	1 (1.1%)	−10.3%	
rtCGM	78 (88.6%)	87 (98.9%)	+10.3%	
Transferred patients (*N* = 50)				
MDI	6 (12%)	2 (4%)	−8%	
SAP	10 (20%)	1 (2%)	−18%	
AID	34 (68%)	47 (94%)	+ 26%	0.002
FGM	0 (0%)	0 (0%)	0%	
rtCGM	50 (100%)	50 (100%)	0%	n.a
Acquired patients (*N* = 38)				
MDI	19 (50%)	8 (21.1%)	−28.9%	
SAP	9 (23.7%)	7 (18.4%)	−5.3%	
AID	10 (26.3%)	23 (60.5%)	+ 34.2%	0.005
FGM	10 (26.3%)	1 (2.6%)	−23.7%	
rtCGM	28 (73.7%)	37 (97.4%)	+23.7%	0.007

Data are reported as absolute numbers with corresponding percentages in parentheses. *p*-values refer to within-group comparisons over time. Statistical testing was not applicable for rtCGM use in transferred patients due to 100% prevalence at both time points. MDI, Multiple Daily Injection; SAP, Sensor Augmented Pump; AID, Automated Insulin Delivery; FGM, Flash Glucose Monitoring; rtCGM, real time Continuous Glucose Monitoring; n.a., not applicable.

### 3.4 Environmental and economic impact

The analysis of the economic and environmental impact of the Hub and Spoke model was carried out on the 88 patients included in the study. Based on standardized travel assumptions, the total number of kilometers saved was estimated at 70192, corresponding to an overall reduction of 11,932 kg of CO₂ emissions and a total estimated travel cost saving of € 25,760. This translated into an estimated average of 797.6 km, 135.6 kg of CO₂, and € 292.7 saved per patient per year (Table 6).

**Table 6:** Environmental impact of the Hub and Spoke system in terms of estimated kilometers, CO2 emissions and travel-related costs saved during the first year of its implementation.

Estimate	Travel (Km)	CO2 emissions (KgCO2)	Costs (€)
Per year	70,192	11,932	25,760
Per patient / Per year	797.6	135.6	292.7

Calculations are made on the 88 patients included in the study.

## 4 Discussion

This study describes the implementation and evaluation of an innovative regional care model for pediatric T1D, based on a Hub and Spoke system that integrates a central tertiary care center with multiple peripheral sites. Classical descriptions of the Hub and Spoke system highlight its reliance on centralization, with advanced expertise and technologies located at the hub and peripheral spokes acting mainly as referral sites for complex cases (6). This organization aims to optimize efficiency through resource concentration and to ensure high specialization within the hub. However, in our regional application the model was adapted with the explicit goal of disseminating protocols, training, and technological support from the hub to peripheral centers, ensuring more consistent, efficient, and accessible diabetes care throughout the region, while preserving the hub’s role in managing the most complex cases. In our experience, challenges typically associated with Hub and Spoke systems were turned into advantages. Decentralizing routine follow-up to peripheral centers prevented hub congestion, while bringing care closer to families reduced travel distance, time, costs, and CO₂ emissions. When considering the entire population currently followed within our Hub and Spoke system, the magnitude of these benefits becomes even more evident. Extending the per-patient annual savings estimated in our study to all 129 patients currently in care within the system translates into approximately 102,000 km of travel, 17,340 KgCO₂ and €37,434 saved annually. This is particularly relevant in Liguria, a region characterized by a single congested highway and railway connecting the provinces to Genoa, and where over 50% of transportation is ensured by private car (15). Importantly, our study confirms not only the organizational, but also the clinical impact of the model. Over 12 months, we observed a progressive increase in the adoption of advanced technologies, especially AID systems, within an organizational framework that facilitated their uptake, with downstream effects on glycemic control. These improvements are clinically meaningful, as TIR is increasingly recognized as the key indicator of overall glycemic control and a strong predictor of long-term outcomes (16). Although HbA1c remains widely used for long-term metabolic assessment, CGM-derived metrics were prioritized in this study, as they provide a more comprehensive and clinically actionable evaluation of glycemic control (17). Evidence from large cohorts show that each 10% increase in TIR corresponds to a substantial reduction in the risk of retinopathy, nephropathy, and neuropathy (18). Moreover, these improvements were not limited to changes in mean CGM values but also translated into a higher proportion of patients achieving international recommended glycaemic targets (8). The role of AID in improving outcomes is well documented: in a meta-analysis of over 100,000 participants, AID use was associated with a mean TIR increase of 11.6%, consistent across age groups and follow-up durations (19). Interestingly, the greatest improvements and size effects in glycemic control were observed in the subgroup of acquired patients, compared to transferred. This can be largely attributed to their transition to specialized pediatric follow-up, which ensured the application of updated standardized protocols, easier access to multidisciplinary support, and ultimately facilitated the adoption of advanced technologies such as AID. Notably, at 12 months, acquired patients achieved a higher absolute TIR than transferred patients despite a lower overall use of AID. This could in part be explained by a time-on-therapy effect: a greater proportion of acquired patients initiated AID during the study period, thus benefiting from the early effects of these systems, whereas transferred patients, who already had higher baseline use, may have experienced a partial attenuation of these benefits over time (20). In addition to conventional CGM metrics, TITR and GRI were included in our analysis. TITR has been proposed as a key marker of optimal glycemic control in the era of advanced technologies (21), while GRI provides a single-number summary of both hypo- and hyperglycemia risk (22). The observed improvements in both metrics further support the positive impact of the Hub and Spoke model on overall glycemic quality and stability. Alongside the overall improvement in glycemic control, an increase in hypoglycemia exposure was observed, more pronounced among acquired patients. This pattern contrasts with most randomized trials and meta-analyses of AID systems, which generally do not report significant variation in time spent in hypoglycemia after AID initiation, and therefore warrants careful clinical interpretation (23). Particular attention is given to hypoglycemia risk during follow-up, and the observed increase was addressed by reinforcing therapeutic education, with specific focus on insulin dosing, carbohydrate counting, and hypoglycemia prevention strategies, as well as by closer clinical monitoring. Ongoing follow-up will be essential to determine whether this pattern attenuates over time. The effectiveness of a Hub and Spoke model has already been demonstrated in Liguria for the management of pediatric celiac disease, showing its positive impact on uniformity of care and reduction of travel burden (15). Our findings extend this evidence to pediatric T1D. In addition, a previous work in the T1D field suggested that Hub and Spoke approaches can be feasible and beneficial, even if applied only to structured therapeutic education, reporting improvements in glycemic outcomes and reduced acute complications while ensuring equitable access across centers (24). Our results suggested that Hub and Spoke models can be successfully adapted to comprehensive diabetes management.

A major strength of this study is that it provides the first comprehensive evaluation of a Hub and Spoke model applied to the full spectrum of pediatric and young adult T1D care. The initiative was implemented region-wide, including all pediatric departments, and was supported by shared governance and standardized protocols. The systematic collection of clinical and organizational data, along with the estimation of environmental and economic impacts, allowed for a multidimensional assessment of the model. Another strength is the use of advanced glycemic metrics, including time in tight range and glycemia risk index, which provide a more granular understanding of the improvements observed.

This study also has several limitations. First, its observational design and the absence of an external control group limit causal inference, as part of the observed improvements may reflect general trends, including the progressive national dissemination of AID systems, which may have contributed to increased technology uptakeSecond, although CGM-derived metrics were intentionally prioritized, not all patients used the same glucose monitoring system, and laboratory-based outcomes such as HbA1c were not consistently available for the entire cohort, which may affect comparability with other studies. Third, baseline differences between transferred and acquired patients, particularly in glycemic control and technology use, may have influenced subgroup analyses and effect size estimates thereby limiting the interpretability of between-group comparisons. Fourth, information on healthcare resource utilization, such as outpatient visit frequency, was incomplete at baseline for acquired patients previously followed outside the network, limiting longitudinal comparisons for this outcome. Fifth, although the model was implemented across the entire region, its generalizability may be influenced by the specific organizational context of Liguria, characterized by centralized governance within a single IRCCS network and distinct geographic features. In addition, heterogeneity among the spoke centers in terms of local resources, staff availability, or organizational capacity may have contributed to variability in implementation and outcomes across the network. Sixth, the duration of follow-up was limited to 1 year, and longer-term data will be required to assess the durability of clinical, organizational, and technological effects. Seventh, although the study included all pediatric patients in the region, the absolute sample size remains relatively small compared with large international cohorts, and some secondary analyses may have been underpowered. Eighth, estimates of kilometers, travel-related costs and CO₂ emissions were derived from standardized assumptions and reference values and should be interpreted as approximations rather than precise individual savings. Finally, qualitative data on patient and provider experience were not yet available at the time of this analysis. The evaluation of satisfaction among families and healthcare professionals in peripheral centers is currently ongoing and will be reported in future publications.

## 5 Conclusion

The implementation of a regional Hub and Spoke model for pediatric T1D care in Liguria proved to be a feasible and effective strategy for delivering equitable, standardized and high-quality care across a geographically complex region. Over the first year, the model was associated with significant improvements in glycemic control, particularly among patients not previously managed by specialized centers. These improvements paralleled increased adoption of advanced diabetes technologies, notably rtCGM and AID systems. Beyond clinical outcomes, the model also demonstrated organizational and societal benefits, including reduced travel burden for families, lower associated costs, and decreased environmental impact. The structured integration of peripheral centers into a coordinated network, supported by shared protocols and regular specialist collaboration, enabled the delivery of specialized care closer to home while maintaining high standards of quality. Furthermore, the model appeared especially beneficial for patients newly integrated into the network, supporting its role in reducing healthcare disparities and promoting equity of access. Taken together, these findings suggest that a Hub and Spoke model, when supported by appropriate governance and resource allocation, can serve as a scalable and sustainable framework for chronic disease management in pediatric populations including settings that ensure continuity of care beyond adolescence. Future work should focus on long-term outcomes, cost-effectiveness analyses, and qualitative assessments to further evaluate the impact of this model on patients and providers experience.

## Data Availability

The original contributions presented in the study are included in the article/Supplementary material, further inquiries can be directed to the corresponding author.
