# Supplementary material for: A region-wide hub and spoke approach to standardize and decentralize type 1 diabetes management
Source: Front Public Health. 2026 Feb 11;14:1763175. doi: 10.3389/fpubh.2026.1763175 (PMC12932541; doi:10.3389/fpubh.2026.1763175)
Supplement: Supplementary file 1 [file Data_Sheet_1.pdf]

1. INDICE

1. INDICE 1
2. STORIA DEL DOCUMENTO 2
3. SCOPO E CAMPO DI APPLICAZIONE....3
4. DEFINIZIONI,ACRONIMI E ABBREVIAZIONI....3
5. MODALITA' OPERATIVE E RESPONSABILITA'....4
   5.1 Compiti del Centro di Riferimento Regionale....4
   5.2 Compiti dei Presidi della Rete del "Gaslini Diffuso" 4
   5.3 Risultati attesi....5
   5.4 Protocollo operativo nelle S.C. Pediatria del Gaslini Diffuso....5
6. DISTRIBUZIONE E ARCHIVIAZIONE....7
7. RIFERIMENTI....7
8. ALLEGATI....8

Questo documento è di proprietà dell'IRCCS "GIANNINA GASLINI", ogni riproduzione non autorizzata dallo stesso è vietata

2. STORIA DEL DOCUMENTO

Rev.	Data	Redazione	Revisione	Verifica	Approvazione	Modifiche
0	12/09/2023	Elio Castagnola<br>Dipartimento di Scienze Medico-Pediatriche<br><br>Mohamad Maghnie<br>Nicola Minuto<br>U.O.C. Clinica Pediatrica - Endocrinologia<br><br>Riccardo Borea<br>Alberto Gaiero<br>Maria Franca Corona<br>Andrea Gazzolo<br>S.C. Pediatrie Gaslini Diffuso	Marisa Alberti<br>Direzione Sanitaria<br><br>Roberta Tirone<br>RAD Pediatria<br><br>Ines Lorenzi<br>UOC Controllo di Gestione, Qualità e Sicurezza	Giuseppe Spiga<br>RSGQ	Raffaele Spiazzi<br>Direttore sanitario<br><br>FIRMATO DIGITALMENTE:<br>Direttore Sanitario -<br>Raffaele Spiazzi<br>(19/01/2024 11:59)<br>[firma_digitale]	Prima emissione

Questo documento è di proprietà dell'IRCCS "GIANNINA GASLINI", ogni riproduzione non autorizzata dallo stesso è vietata

3. SCOPO E CAMPO D'APPLICAZIONE

Scopo del percorso è definire un iter diagnostico-terapeutico assistenziale per la gestione del paziente con diabete nell'ambito del Gaslini Diffuso.

Le Unità Operative coinvolte sono la U.O.C. Clinica Pediatrica-Endocrinologia dell'IRCCS Istituto Giannina Gaslini quale Centro di Riferimento Regionale e le SS.CC. Pediatria del "Gaslini Diffuso".

Sarà trattato il Diabete Mellito di tipo 1 (DM1) per cui sarà assicurato l'inquadramento diagnostico, la presa in carico multidisciplinare, la terapia, il follow-up e le attività di formazione-informazione-educazione rivolte alle famiglie e agli operatori sanitari.

4. DEFINIZIONI, ACRONIMI E ABBREVIAZIONI

Diabete Mellito (DM): malattia cronica caratterizzata da iperglicemia e dovuta ad una alterata secrezione di insulina. Si distinguono due tipologie principali di diabete: (i)diabete mellito di tipo 1 (DM1) che esordisce principalmente nei bambini-adolescenti, (ii) diabete mellito di tipo 2 (DM2) più tipico dell'adulto. L'International Diabetes Federation (IDF) nel 2021 stima che vi siano 1,2 milioni di bambini e adolescenti (0- 19 anni) affetti da DM1 nel mondo e che 536,6 milioni di persone tra 20 e 79 anni (9,8% degli adulti) siano diabetiche.

La patologia diabetica può comportare numerose complicanze: microangiopatiche e macroangiopatiche.

La qualità dell'assistenza e la gestione territoriale della patologia rivestono grande importanza ai fini di un miglior controllo della malattia e della prognosi delle complicanze.

Hub/Spoke: Istituto centrale (definito *Hub*) a cui afferiscono i poli periferici (detti *Spoke*).

Linee Guida: raccomandazioni di comportamento clinico, prodotte attraverso un processo di revisione sistematica della letteratura e delle opinioni degli esperti, elaborate allo scopo di supportare i professionisti nel decidere quali siano le modalità di assistenza più appropriate in specifiche circostanze cliniche.

Procedura o istruzione operativa: definisce la sequenza di azioni tecnico-operative eseguite dal professionista e rappresenta l'unità elementare del processo assistenziale descritta in un documento nel quale viene spiegato analiticamente come si svolge il processo.

Questo documento è di proprietà dell'IRCCS "GIANNINA GASLINI", ogni riproduzione non autorizzata dallo stesso è vietata

5. MODALITA' OPERATIVE E RESPONSABILITA'

Nell'ottica del progetto "Gaslini Diffuso" il modello di assistenza sarà a gestione integrata dei centri hub&spoke, al fine di garantire un'assistenza uniforme ed efficiente su tutto il territorio ligure per i pazienti pediatrici con nuova diagnosi di diabete e per la prevenzione e lo screening delle complicanze e patologie autoimmuni associate al DM1nell'ambito del follow-up longitudinale.

5.1 Compiti del Centro di Riferimento Regionale

- Tutti i compiti dei Presidi della Rete del "Gaslini Diffuso"
- Attività clinica rivolta all'educazione terapeutica sia in Istituto mediante l'impiego dei più innovativi strumenti tecnologici, sia esternamente, tramite diffusione della conoscenza del DM1 nelle scuole, organizzazione di campi di istruzione, di Congressi e Corsi di aggiornamento
- Applicazione del microinfusore ai pazienti provenienti dai poli del Gaslini Diffuso per prosecuzione della terapia insulinica
- Programmazione dei controlli e cambio set dei microinfusori, da concordare con il centro spoke se eseguiti in consulenza da remoto o in autonomia a livello ambulatoriale del Gaslini Diffuso
- Consulenza clinica per i Presidi della Rete del "Gaslini Diffuso" anche in modalità Telehealth
- Verifica dell'adeguatezza e dell'aderenza alle Linee Guida per la diagnosi e il follow-up del diabete nell'ambito del progetto "Gaslini Diffuso"
- Omogeneizzazione e monitoraggio dell'assistenza regionale del "Gaslini Diffuso" in ambito di diabete
- Periodiche iniziative di aggiornamento e formazione del personale del "Gaslini Diffuso"
- Periodiche visite ambulatoriali presso i Presidi del "Gaslini Diffuso" degli specialisti del centro di riferimento regionale con affiancamento dei colleghi del polo
- Formazione dei colleghi individuati nelle SS.CC. Pediatria del Gaslini Diffuso dove è presente attività ambulatoriale sia in Sede che presso i Presidi

5.2 Compiti dei Presidi della Rete del "Gaslini Diffuso"

- Inquadramento iniziale del paziente con sospetto diabete e completamento del percorso diagnostico
- Stabilizzazione paziente, trattamento secondo il protocollo di gestione della chetoacidosi diabetica e avviamento della terapia insulinica
- Applicazione del sensore glicemico

Questo documento è di proprietà dell'IRCCS "GIANNINA GASLINI", ogni riproduzione non autorizzata dallo stesso è vietata

- Informazioni sulla gestione delle emergenze diabetologiche e relativi contatti
- Programmazione ed esecuzione dei follow-up dei pazienti per la sorveglianza della terapia e prevenzione dell'insorgenza di complicanze
- Invio del caso clinico al Centro di Riferimento Regionale in caso di complicanze/sviluppo di patologie associate o per il DH annuale
- Educazione e informazione continua ai pazienti ed ai rispettivi nuclei familiari
- Collaborazione con Pediatri di Libera Scelta per la condivisione dei pazienti diabetici in follow-up
- Periodiche iniziative di aggiornamento e formazione dei pediatri di libera scelta e del personale del presidio

5.3 Risultati attesi

- Miglioramento dell'assistenza al paziente diabetico dalla diagnosi al follow-up mediante omogeneizzazione del percorso diagnostico-terapeutico
- Territorializzazione dell'attività ambulatoriale riducendo in questo modo le liste di attese e le distanze per le famiglie per l'accesso alle cure
- Condivisione delle Linee Guida e raccomandazioni licenziate dalla Società Scientifiche nonchè dei casi complessi migliorando l'efficienza dell'assistenza e riducendo il rischio di non uniformità
- Avviare un percorso di integrazione per redigere un PDTA e per riorganizzare l'assistenza per la fascia di popolazione diabetica nella fase di transizione da adolescente ad adulto, che corrisponde al passaggio di presa in carico da PLS- pediatra-diabetologo a MMG- diabetologo dell'adulto.

5.4 Protocollo operativo nelle S.C. Pediatria del Gaslini Diffuso

Dopo il ricovero che ha portato alla nuova diagnosi di diabete, il paziente viene indirizzato al DH/Ambulatorio di competenza per la presa in carico.

Responsabilità, tempi e contenuti delle attività assistenziali rese ai pazienti e alle loro famiglie nella fase di esordio ai fini della presa in carico degli stessi sono definite nello specifico PDTA e nei protocolli operativi a questo collegati.

Le attività assicurate dal DH/Ambulatorio prevedono:

- educazione e formazione della famiglia
- terapia
- monitoraggio glicemico
- gestione del DM nella vita quotidiana

Questo documento è di proprietà dell'IRCCS "GIANNINA GASLINI", ogni riproduzione non autorizzata dallo stesso è vietata

- gestione ipoglicemia e ipoglicemia severa
- malattie intercorrenti
- gestione del diabete a scuola
- compilazione pratiche 104
- presentazione dei progetti in essere

L'attività assistenziale di diabetologia viene assicurata presso i Presidi del Gaslini Diffuso, sia con la presenza degli specialisti dell'Istituto, sia da remoto in forma di consulenza, affiancamento, telemedicina.

L'attività ambulatoriale di visita diabetologica (circa 3 visite annuali per paziente) viene svolta nei poli del Gaslini Diffuso dallo specialista dell'Istituto con una frequenza generalmente settimanale a Savona e bimestrale a Imperia o La Spezia.

In tali sedi viene garantita la presenza di una postazione computer collegata alla rete VPN Gaslini per consentire l'accesso ai programmi informatici utili per l'attività clinica e la gestione dell'agenda di appuntamenti ("Soldanella", "Galileo", "Aurora" e "Smart Digital").

Il medico diabetologo compila la richiesta, ossia la ricetta dematerializzata, per la visita e si occupa di prenotare l'appuntamento nella relativa agenda disponibile sul programma Soldanella.

Successivamente, dopo aver effettuato la visita, il medico deve inoltre contrassegnare all'interno dello stesso programma la prestazione come "erogata".

Oltre alla visita ambulatoriale, è possibile eventualmente ricorrere alle prestazioni di telemedicina per le attività di visita e di monitoraggio.

L'attività di ricovero diurno (Day Hospital-DH) viene invece attuata nella sede di Istituto a Genova.

Presso il DH afferiscono, idealmente una o massimo due volte all'anno, tutti i pazienti seguiti per DM1, per screening patologie associate, eventuale avvio microinfusore, complicanze e valutazione annuale dietologica e psicologica nonché la partecipazione ai progetti di ricerca.

Ulteriore supporto e assistenza ai pazienti ed alle loro famiglie viene offerto grazie alla collaborazione con le Associazioni che si occupano di diabete in età giovanile.

È prevista con le famiglie la condivisione delle diverse iniziative organizzate con il supporto delle Associazioni, quali a titolo di esempio la formazione nelle scuole del personale docente e di supporto o la possibilità di partecipare ai campi scuola rivolti ai pazienti.

Questi eventi durano una settimana e prevedono la partecipazione di 25-35 pazienti seguiti da tutti gli ambulatori del Gaslini in generale tra 6 e 16 anni.

Nel corso degli anni, si sono anche organizzati eventi più brevi che prevedevano la partecipazione di:

- Famiglie di pazienti sotto i sei anni (con genitori e fratelli)

Questo documento è di proprietà dell'IRCCS "GIANNINA GASLINI", ogni riproduzione non autorizzata dallo stesso è vietata

- Adolescenti – giovani adulti 16-25 anni
- Corsi per formazione pazienti e famiglie guida

6. DISTRIBUZIONE E ARCHIVIAZIONE

La procedura è pubblicata nell'Archivio documentale del Sistema qualità aziendale dell'Istituto e ne viene data comunicazione mediante mail inviata a tutto Istituto.

7. RIFERIMENTI

- Relazione al Parlamento sullo stato delle conoscenze e delle nuove acquisizioni in tema di diabete mellito - Legge 16 marzo 1987, n. 115, recante Disposizioni per la prevenzione e la cura del diabete mellito - Anno 2021 https://www.salute.gov.it/imgs/C_17_pubblicazioni_3229_allegato.pdf
- Regione Liguria. Legge Regionale 9 Agosto 2013, n.27. Norme per la prevenzione, la diagnosi e la cura del diabete mellito
- ISPAD Clinical Practice Consensus Guidelines 2022: The delivery of ambulatory diabetes care to children and adolescents with diabetes. Limbert C, Tinti D, Malik F, et al. Pediatr Diabetes. 2022 Dec;23(8):1243-1269. doi: 10.1111/pedi.13417. PMID: 36537530.
- ISPAD Clinical Practice Consensus Guidelines 2022: Diabetes education in children and adolescents. Lindholm Olinder A, De Abreu M, Greene S, et al. Pediatr Diabetes. 2022 Dec;23(8):1229-1242. doi: 10.1111/pedi.13418. PMID: 36120721; PMCID: PMC10107631.
- ISPAD Clinical Practice Consensus Guidelines 2022: Glycemic targets and glucose monitoring for children, adolescents, and young people with diabetes. deBock M, Codner E, Craig ME, et al. Pediatr Diabetes. 2022 Dec;23(8):1270-1276. doi: 10.1111/pedi.13455. PMID: 36537523; PMCID: PMC10107615.
- ISPAD Clinical Practice Consensus Guidelines 2022: Diabetes technologies: Insulin delivery. Sherr JL, Schoelwer M, Dos Santos TJ, et al. Pediatr Diabetes. 2022 Dec;23(8):1406-1431. doi: 10.1111/pedi.13421. Epub 2022 Dec 5. PMID: 36468192.
- ISPAD Clinical Practice Consensus Guidelines 2022: Microvascular and macrovascular complications in children and adolescents with diabetes. Bjornstad P, Dart A, Donaghue KC, et al. Pediatr Diabetes. 2022 Dec;23(8):1432-1450. doi: 10.1111/pedi.13444. PMID: 36537531.
- PDTA per la gestione ed il trattamento del paziente con diabete in età pediatrica, presente sul Sistema Qualità Aziendale dell' IIGG.

Questo documento è di proprietà dell'IRCCS "GIANNINA GASLINI", ogni riproduzione non autorizzata dallo stesso è vietata

- Proceduura Specifica "PROS- PS-084 Trattamento del paziente con diabete mellito neo diagnosticato di tipo 1 in chetoacidosi in ps e medicina d'urgenza", presente sul Sistema Qualità Aziendale dell' IIGG.

8. ALLEGATI

La procedura non prevede allegati

Questo documento è di proprietà dell'IRCCS "GIANNINA GASLINI", ogni riproduzione non autorizzata dallo stesso è vietata
