# Supplementary material for: A region-wide hub and spoke approach to standardize and decentralize type 1 diabetes management
Source: Front Public Health. 2026 Feb 11;14:1763175. doi: 10.3389/fpubh.2026.1763175 (PMC12932541; doi:10.3389/fpubh.2026.1763175)
Supplement: Supplementary file 2 [file Table_1.docx]

**Supplementary Table 1.** Baseline characteristics of new-onset patients (N=27). Data are presented as mean ± SD or as n (%), unless otherwise specified.

|  | T0 – Baseline (N=27) |
| --- | --- |
| Sex assigned at birth |  |
| Female  Male | 18 (66.7%)  9 (33.3%) |
| Ethnicity |  |
| African  Hispanic  Caucasic | 6 (22.2%)  1 (3.7%)  20 (74.1%) |
| Age at T1D onset (years) | 7.50 ± 4.22 |
| Follow-up spoke center |  |
| Imperia  La Spezia  Savona | 9 (33.3%)  8 (29.6%)  10 (37.1%) |

**Supplementary Table 2.** Baseline characteristics of all transferred and acquired patients (N=102). Data are presented as mean ± SD or as n (%), unless otherwise specified.

|  | **T0 – Baseline (N=102)** |
| --- | --- |
| **Sex assigned at birth** |  |
| Female  Male | 47 (46.1%)  55 (53.9%) |
| **Ethnicity** |  |
| Asiatic  African  Hispanic  Caucasic | 1 (1.0%)  18 (17.6%)  2 (2.3%)  81 (79.4%) |
| **Previous care setting** |  |
| Transferred  Acquired | 60 (58.8%)  42 (41.2%) |
| **Current spoke center** |  |
| Imperia  La Spezia  Savona | 37 (36.3%)  30 (29.4%)  35 (34.3%) |
| **Age (years)** | 13.26 ± 5.96 |
| **Disease duration (years)** | 6.21 ± 5.83 |
| **BMI** | 20.97 ± 4.59 |
| **BMI z-score** | 0.48 ±1.08 |
| **Type of insulin treatment** |  |
| MDI  SAP  AID | 31 (30.4%)  19 (18.6%)  52 (51.0%) |
| **Type of glycemic monitoring** |  |
| FGM  Rt-CGM | 11 (10.8%)  91 (89.2%) |
| **Visits in the previous 12 months*** | 5.19 ± 1.99 |
| **SH episodes in the previous 12 months*** | 0 |
| **DKA episodes in the previous 12 months*** | 0 |

*Data available at T0 only for transferred patients.

List of abbreviations: BMI, body mass index; HbA1c, glycated hemoglobin; MDI, multiple daily injections; SAP, sensor augmented pump; PLGS, pre-low glucose suspend; AID, automated insulin delivery; FGM, flash glucose monitoring; rtCGM, real time continuous glucose monitoring; DKA, diabetic ketoacidosis; SH, severe hypoglycemia.
